# Modeling hemoglobin and hemoglobin:haptoglobin complex clearance in a non-rodent species–pharmacokinetic and therapeutic implications

**DOI:** 10.3389/fphys.2014.00385

**Published:** 2014-10-09

**Authors:** Felicitas S. Boretti, Jin Hyen Baek, Andre F. Palmer, Dominik J. Schaer, Paul W. Buehler

**Affiliations:** ^1^Division of Veterinary Internal Medicine, School of Veterinary Medicine, University of ZurichZurich, Switzerland; ^2^Laboratory of Biochemistry and Vascular Biology, FDA Center for Biologics Evaluation and ResearchBethesda, MD, USA; ^3^Chemical and Biomolecular Engineering, The Ohio State UniversityColumbus, OH, USA; ^4^Division of Internal Medicine, University of Zurich Hospital, University of ZurichZurich, Switzerland

**Keywords:** macrophage uptake, hemoglobin, haptoglobin, non-rodent, pharmacokinetic modeling

## Abstract

**Background:** Haptoglobin (Hp) prevents hemoglobin (Hb) extravasation and attenuates Hb induced tissue oxidation and vasoconstriction. Small animal models such as mouse, rat and guinea pig appear to demonstrate proof-of-concept for Hb neutralization by Hp in diverse pre-clinical conditions. However, these species differ significantly from humans in the clearance of Hb:Hp and demonstrate long persistence of circulating Hb:Hp complexes.

**Objective:** The focus of this study is to understand Hb:Hp clearance in a non-rodent species. In contrast to rodents, dogs maintain high plasma Hp concentrations comparable to humans and demonstrate more rapid clearance of Hb:Hp when compared to rodent species, therefore dogs may represent a relevant species to evaluate Hb:Hp pharmacokinetics and cellular clearance.

**Results:** In this study we show, that like human macrophages, dog peripheral blood monocyte derived macrophages express a glucocorticoid inducible endocytic clearance pathways with a high specificity for the Hb:Hp complex. Evaluating the Beagle dog as a non-rodent model species we provide the first pharmacokinetic parameter estimates of free Hb and Hb:Hp complexes. The data demonstrate a significantly reduced volume of distribution (Vc) for Hb:Hp compared to free Hb, increased maximum plasma concentrations and areas under plasma concentration time curves (Cmax and AUC). Significantly reduced total body clearance (CL) and a longer terminal half-life (t_1/2_) of approximately 12 h were also observed for the Hb:Hp complex. Distribution and clearance were identical for dimeric and multimeric Hb:Hp complexes. We found no significant effect of a high-dose glucocorticoid treatment protocol on Hb:Hp pharmacokinetic parameter estimates.

**Conclusion:** Collectively, our study supports the dog as a non-rodent animal model to study pharmacological and pharmacokinetic aspects of Hb clearance systems and apply the model to studying Hp as a therapeutic in diseases of hemolysis.

## Introduction

Haptoglobin (Hp) therapy may be of benefit during hemolytic states to bind and sequester cell free hemoglobin (Hb) within the vascular compartment (Schaer and Buehler, 2013). Hp decreases the interaction of Hb with critical organ systems and may attenuate free Hb triggered events such as vascular dysfunction and renal injury (Baek et al., 2012). Currently, plasma derived Hp is marketed in Japan with indications for trauma, burn and massive blood transfusion (Schaer et al., 2013). However, several off-label uses of Hp are reported in the literature as individual case studies or small clinical trials in various situations of acute hemolysis (Hashimoto et al., 1993; Yamamoto et al., 2000; Eda et al., 2001). Recent interest within Europe and the US to develop plasma derived Hp as a biologic therapeutic for uses in both acute and chronic hemolytic disease states have been suggested (Schaer et al., 2013). Therefore, understanding the pharmacologic and pharmacokinetic relevance of animal species for predicting human response is a critical aspect of the proof of concept and pre- clinical safety evaluation for Hp therapeutics in early development.

Hp is a α_2_-sialoglycoprotein and the primary Hb-binding protein in plasma. Hp exists in humans as a multi-phenotype protein designated as Hp 1-1, Hp 2-1, and Hp 2-2 (Connell et al., 1962). Normal Hp concentrations in tissue and plasma are within a range between 0.5 and 2 mg/ml (Nosslin and Nyman, 1958; Levy et al., 2010). The dimeric (1-1) and the multimeric forms (2-1 and 2-2) of Hp contain identical β globin chains involved in Hb dimer binding (Andersen et al., 2012), but differ in their α globin chains, which contain either one cysteine (α1) or two cysteines (α2) that are involved in disulfide bond formation (Connell et al., 1962; Smithies et al., 1962a,b). Therefore, the multi-phenotype designation is dependent on the proteins α1 and α2 content (Connell et al., 1962). Conflicting reports either do or do not support specific protective properties of Hp phenotypes (Lipiski et al., 2013; Asleh et al., 2014).

Clearance of Hb:Hp complexes has been suggested to occur via tissue (spleen and liver) resident macrophages (Kristiansen et al., 2001; Schaer et al., 2007). These cells typically express a high surface concentration of CD163 and maximal heme oxygenase-1 (HO-1) enzymatic capacity (Zwadlo et al., 1987). CD163 is a cell surface protein exclusive to monocytes/macrophages and is the putative 9—domain receptor for binding and internalization of Hb:Hp complexes (Kristiansen et al., 2001). Further detoxification of heme takes place via HO-1 to liberate free iron, which is then bound to the ferritin complex until it is reutilized. Monocyte/macrophage clearance may be saturated when supra-physiological concentrations of Hb:Hp complex result from therapeutic Hp administration during hemolysis and this may lead to extended circulation of Hb:Hp within the vascular space. With interest in development of Hp as a biologic therapeutic it remains important to identify species with a “human-like” functional monocyte/macrophage Hb:Hp clearance system that could serve to model Hb:Hp clearance in humans.

Our rationale for evaluating the dog as a model is based on several aspects of Hb clearance systems observed in canines that appear to approximate humans. For example, in contrast to rodents, human and dog have high basal plasma concentrations of Hp (Boretti et al., 2009). We previously reported that dogs are responsive to up-regulation of Hp following glucocorticoid dosing and this leads to attenuation of the acute effects of Hb exposure (Boretti et al., 2009). In these studies we observed, that glucocorticoid treated dogs exposed to cell free Hb accumulated high intravascular concentrations of Hb:Hp complexes that were cleared within 24 h. Based on these observations we hypothesized that the clearance system for cell free Hb in dogs may approximate that of humans. As a result the dog may estimate the pharmacokinetic response to supra-physiological Hb:Hp exposure and allow for evaluation of the pharmacokinetics of Hb following *in vivo* binding to differing Hp phenotypes.

The present study was designed to (1) characterize the Hb:Hp clearance system of dog macrophages *in vitro* based on the well-defined characteristics of the human system, (2) evaluate the effects of glucocorticoid pre-treatment on the pharmacokinetics of Hb and Hb:Hp complexes in the dog and (3) evaluate the pharmacokinetics of Hb and Hb:Hp complexes following the administration of two pre-clinical grade human Hps purified as dimeric or multimeric phenotype composition.

## Materials and methods

### Materials

Human and dog Hb was prepared using a standardized method previously described (Palmer et al., 2009). Human plasma fractionated Hp predominantly in the dimeric form (Hp 1-1) was provided by BioProducts Laboratory (Elstree, UK). Human plasma fractionated Hp predominantly in the multimeric forms (Hp 2-1 and Hp 2-2) was provided by CSL Behring (Kankakee, Illinois, USA). Concentrations of Hb and Hp solutions were prepared as 10 and 6 g/dl, respectively. Reducing and non-reducing gels were carried out according to existing protocols outlined by Life Technologies™ Protocols.

### Preparation of dog macrophages

Mononuclear cells were prepared from heparin anti-coagulated beagle dog blood by Ficoll Paque centrifugation at 420 g for 25 min. After washing twice in phosphate buffered saline (PBS) the CD14^+^ monocytes were isolated from the mononuclear cell fraction by magnetic cell separation using anti-CD14 magnetic MACS beads (Miltenyi Biotec) according to the protocol provided by the manufacturer. The isolated CD14^+^ monocytes were cultured on glass coverslips in DMEM medium containing 10% fetal calf serum (FCS) in the presence of human M-CSF (100 ng/ml; Peprotech) and dexamethasone (2.5 × 10^−7^ M; Sigma) as indicated. According to our previous work, this concentration of dexamethasone induces maximum induction of CD163 and Hb:Hp uptake in human macrophages (Schaer et al., 2002). To evaluate the effect of dexamethasone treatment on Hb:Hp endocytosis, macrophage were analyzed after 36 h in culture. We have previously used this protocol to establish the Hb:Hp uptake stimulation activity of glucocorticoid treatment in human PBMC derived macrophages, and the same cell culture protocol provided evidence for robust CD163 expression in mouse peritoneal macrophages (Schaer et al., 2001). To compare endocytosis capacity for human and dog Hb:Hp we used a longer cell culture protocol of 4 day (in the presence of M-CSF and dexamethasone). After longer culture periods macrophages have a more mature phenotype with maximum CD163 expression and high Hb:Hp endocytic capacity (Schaer et al., 2006a; Kaempfer et al., 2011).

### Fluorescent labeling of human and dog Hp

Dog Hp was from Lee Biosolutions (St. Louis, Missouri USA), human Hp of the dimeric Hp 1-1 phenotype was from BPL BioProducs Laboratory (Elstree, UK). Dog and human Hp were diluted in PBS pH 8.0 to a concentration of 4 mg/ml. 2 mg Hp were incubated with 100 μg Alexa Fluor 488 succinimidyl ester (NHS ester; Moelcular Probes) for 1 h in the dark at room temperature. Subsequently, non-bound dye was removed by a PD-10 desalting column (GE Healthcare).

### Measurement of Hb:Hp endocytosis

Endocytosis of Hb:Hp complexes by dog macrophages was measured as reported previously by a modified fluorescent uptake assay (Schaer et al., 2002, 2008). In our previous work we established that labeling of Hb:Hp complexes with Alexa 488 does not significantly affect endocytosis by CD163 (Schaer et al., 2002, 2006b). Dog macrophages were incubated Alexa 488 labeled dog or human Hp ± (non-labeled) Hb at a concentration of 10 μg/ml in serum free DMEM medium for 30 min at 5% CO_2_/37°C. After incubation the cells were washed with PBS and fixed with 4% buffered formalin for 15 min. Nuclei were stained with Hoechst 33342 DNA stain. From each sample (cover slip) 20 random wide-field fluorescence images were acquired with a Zeiss Observer.Z1 microscope equipped with an Axiocam MRm camera and ZEN 2012 software (Carl Zeiss AG) at an optical magnification of 400x. All image acquisition parameters were set at fixed values for the whole study and no further image processing was applied before quantification. Digital image analysis was performed using Cell Profiler image analysis software (Broad Institute; www.cellprofiler.org) to (1) identify and count individual cells in the Hoechst nuclear stain channel and (2) to quantify Alexa488 fluorescence intensity per cell in the green channel image. Subtracted background values were determined with macrophage samples that have not been incubated with a fluorescent protein. Images for illustration were acquired as Z-stack optical section images from the identical samples using an Apotome.2 image acquisition system at an optical magnification of 400x (Carl Zeiss AG). The final images were composed using Fiji/ImageJ software (http://fiji.sc/Fiji) with identical threshold values applied to all images.

### Design of animal studies

All studies were performed in accordance with the Swiss Academy of Medical Sciences ethical principles and guidelines for experiments on animals. The protocol for the study was reviewed and approved by the Swiss Institutional Animal Care and Use Committee as meeting the Swiss guidelines for laboratory animal use. The present study was conducted to evaluate plasma concentration vs. time data and pharmacokinetics following exogenous Hb exposure of 4 g to seven separate groups of beagle dogs. After implantation of a jugular cannula and recovery, conscious Beagle dogs were randomized to 1 of 7 groups as follows: (1) dog Hb, 4 g dose (*n* = 4); (2) human Hb, 4 g dose (*n* = 4); (3) prednisone pre-treated (50 mg/kg, twice daily, 3 days) (Martinez-Subiela et al., 2004) + Dog Hb, 4 g dose (*n* = 6); (4) prednisone pre-treated (50 mg/kg, twice daily, 3 days) + human Hb, 4 g dose (*n* = 6); (5) human Hb 4 g dose + dimeric human Hp, 4 g dose (*n* = 6); (6) human Hb 4 g dose + multimeric human Hp, 4 g dose (*n* = 6) and (7) prednisone pre-treated (50 mg/kg, twice daily, 3 days) (Martinez-Subiela et al., 2004) + human Hb, 4 g dose + multimeric Hp, 4 g dose (*n* = 6). Dosing regimen: Animals were first dosed over a 10 min period with a 4 g bolus of Hb (40 ml, concentration 10 g/dl) followed immediately by a 4 g dose of Hp dimeric or multimeric (67 ml, concentration 6 g/dl) administered over a 10 min period. Whole blood (1 ml) per sample was obtained from the jugular vein at baseline and post Hb and Hp dosing: 5 min., 0.25, 0.5, 0.75, 1.0, 1.5, 2.0, 2.5, 3, 3.5, 4.0, 5.0, 6.0, 8.0, 12, 16, 24, 32, 36, 48 and 60 h. After the initial 24 h of blood collections, the jugular cannula was removed and blood sampling until 60 h was performed using a 21 gauge needle and syringe. After the 60 h collection animals were returned to their colony. Whole blood was collected in heparinized 3 ml syringes, centrifuged at 4000 rpm for 10 min and plasma was frozen at −80°C until uv-visible spectrophotometry and size exclusion-high performance liquid chromatography analysis. The speed of centrifugation did not lead to hemolysis. All basal and final collection blood samples were free of extra-cellular Hb based on visual and spectral analysis.

### Determination of plasma Hb concentrations

Plasma collected at baseline from each animal served as the blank to correct for background interference and turbidity. Ferrous heme (oxy/deoxy), ferric heme, and hemichrome were determined on photodiode array spectrophotometer (Model 8453 Hewlet Packard, Palo Alto, CA). Spectral data was evaluated using a multi-component analysis based on the extinction coefficients for each species and total heme was calculated by adding these values (Winterbourn, 1985). The Plasma concentration time data are represented as plasma heme micro-molar concentration bound within Hb or Hb:Hp vs. time in hours. Free and Hp-bound Hb were determined in plasma (50 μL) by size exclusion chromatography (SEC) run on a BioSep-SEC-S3000 (600 × 7.5 mm) SEC column (Phenomenex, Torrance, CA) attached to a Waters Delta 600 pump and Waters 2499 dual-wavelength detector, controlled by a Waters 600 controller using Empower™ 2 software (Waters Corp., Milford, MA). Peak areas for Hb and Hb:Hp obtained at 405 nm were recorded. Calculations for percent free Hb were determined as follows (Hb peak area/total Hb + Hb:Hp peak areas) × 100 = percent free Hb. Heme concentrations from uv-visible spectrometry x percent free Hb = free Hb concentration (as μM heme). Similarly, Hb bound to Hp was calculated as follows (Hb:Hp peak area/total Hb + Hb:Hp peak areas) ×100 = percent Hb bound to Hp (Hb:Hp). Heme concentrations from uv-visible spectrometry × percent Hb:Hp = Hb concentration (as μM heme) bound to Hp (Hb:Hp).

### Pharmacokinetic parameter estimate evaluation

A non-compartmental pharmacokinetic analysis was performed on unbound Hb (group 1) and Hb bound to Hp (groups 2 and 3). Plasma Hb concentrations using WinNonlin version 5.2.1, Pharsight company (St. Louis, Missouri). Pharmacokinetic estimates are expressed in tabular form as mean ± SEM. Maximal plasma concentrations (Cmax) represent the greatest plasma concentration reached after dosing in each animal. Area under the plasma concentration time curve (AUC_0−inf_) was estimated using the linear trapezoidal rule to the last measurable concentration (AUC_0−Clast_) where C_last_ is the last measurable plasma concentration. Extrapolation to infinity (AUC_Clast−inf_) was calculated by dividing C_last_ by the negative value of the terminal slope (*k*) of the log-linear plasma concentration-time curve. Thus, AUC_0−∞_ is equal to the sum of AUC_0−Clast_ and AUC_Clast−inf_. Additional parameters were calculated as follows: the plasma clearance (CL) was calculated as the dose divided by AUC_0−inf_, half-life (t_1/2_) was calculated as ln(2) ^*^ Vc/CL. Volumes of distribution of the central compartment (Vc) are calculated as the product of dose (as heme equivalents) divided by Cmax.

## Results

### Characterization of the dog monocyte/macrophage Hb:Hp complex clearance system

**Figure 1:**
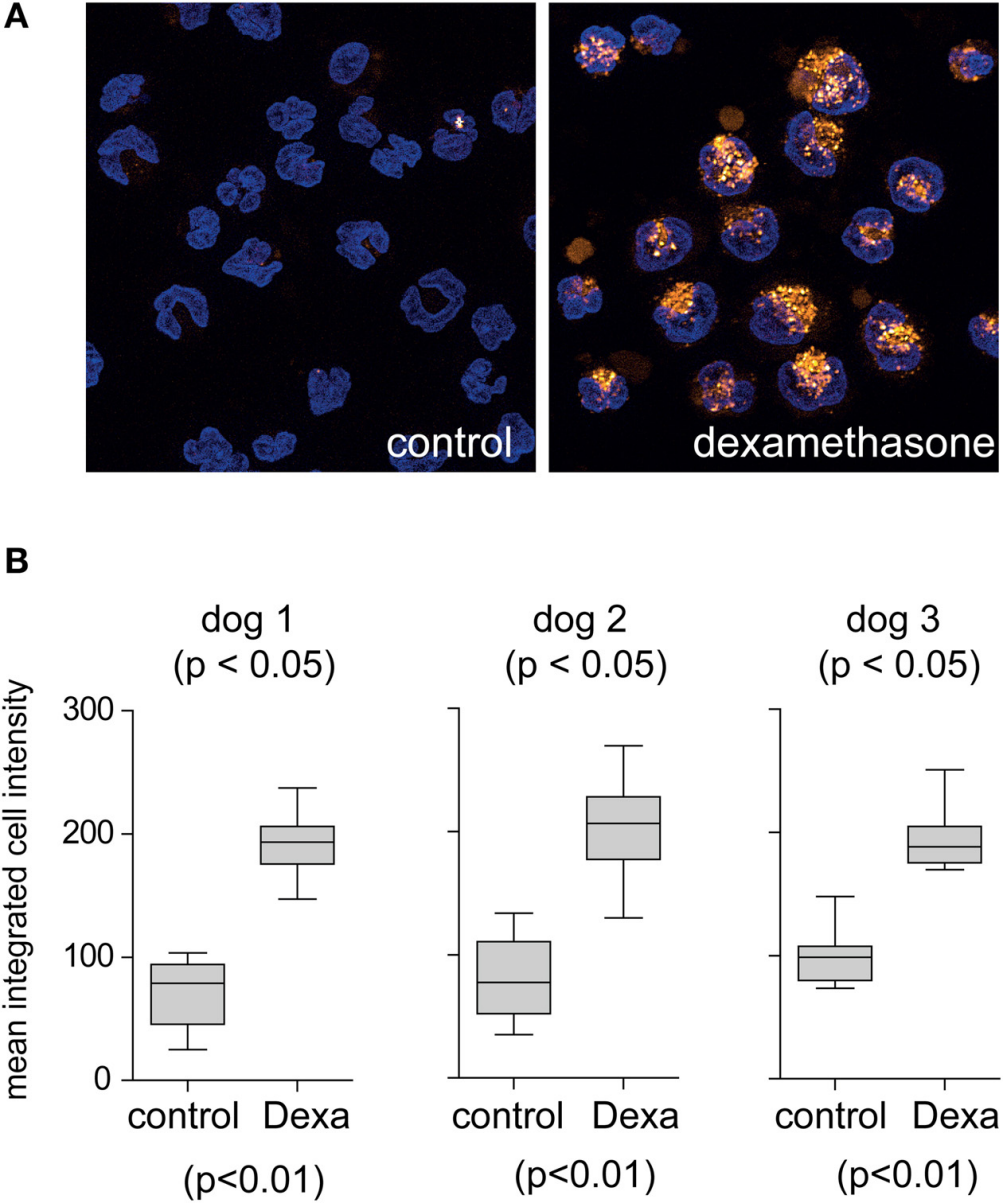
**Glucocorticoid induction of the dog macrophage Hb:Hp clearance system. (A)** Peripheral blood CD14^+^ monocytes from beagle dogs were cultured for 36 h in the presence of M-CSF (control) or M-CSF + dexamethasone (2.5 × 10^−7^ M) before they were incubated with fluorescent dog Hb:Hp complexes for 30 min. Images represent maximum orthogonal projections of z-stack images acquired with an Apotome fluorescence microscopy system. Blue: nuclei; orange: Hb:Hp complex. Original optical magnification 400x. **(B)** Hb:Hp upake was quantified by digital image analysis in control and dexamethasone treated macrophages from three different beagle dogs, each condition is represented by 15 individual random images.

**Figure 2:**
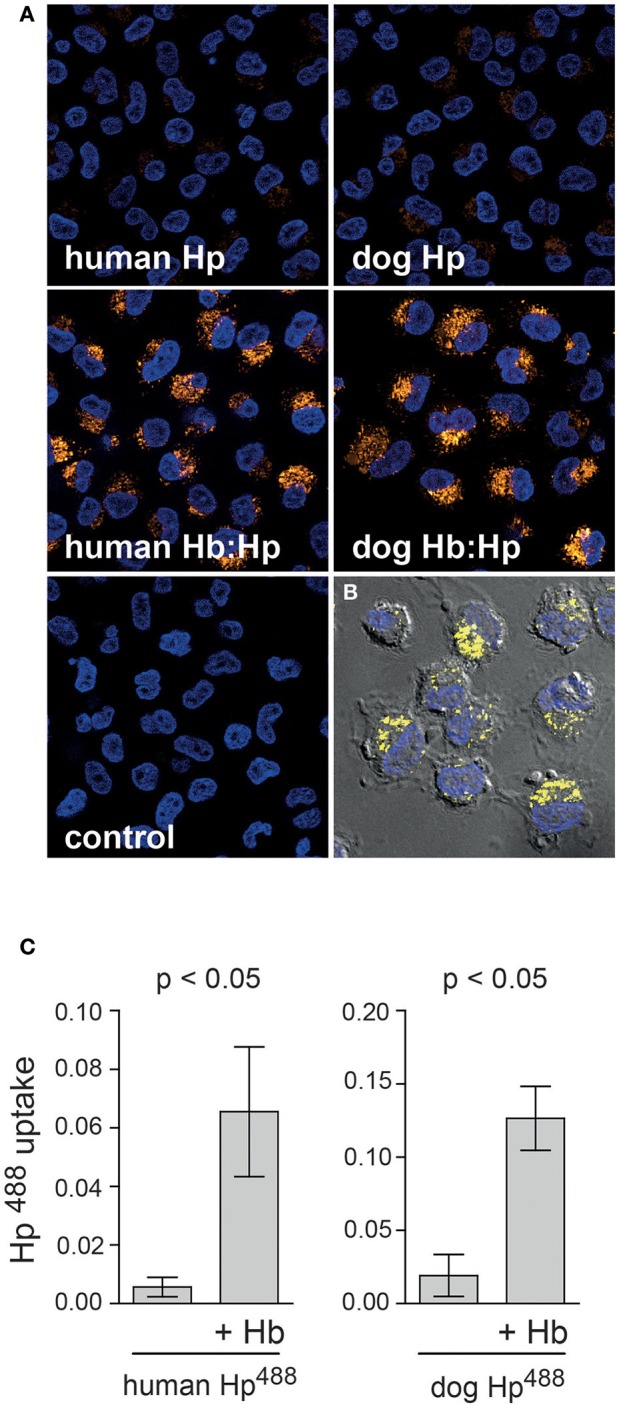
**Specificity of the dog macrophage clearance system for Hb:Hp complexes. (A)** Peripheral blood CD14^+^ monocytes from beagle dogs were cultured for 4 days in the presence of M-CSF + dexamethasone (2.5 × 10^−7^ M) before they were incubated with fluorescent (Alexa488) dog or human Hp with or without Hb for 30 min. Images are single optical sections acquired with an Apotome fluorescence microscopy system. Blue: nuclei; Orange: Hb:Hp complex. Original optical magnification 400x. **(B)** Fluorescence signals of a single optical apotome section as shown in **(A)** overlaid on a DIC bright-field image, which indicates cell boarders and intracellular localization of the endocytosed Hb:Hp complexes in the perinuclear region of the macrophages. Blue: nuclei; Yellow: Hb:Hp complex. **(C)** Dog and human Hp ± Hb uptake was quantified by digital image analysis in macrophages from five different beagle dogs.

To characterize the dog monocyte/macrophage system for clearance of Hb:Hp complexes and to evaluate the potential validity of this animal model for studies with human Hp we have investigated two essential functions that are characteristic for the human monocyte/macrophage Hb:Hp clearance system. These characteristics are the glucocorticoid responsiveness of the human system and the specificity for uptake of Hb:Hp complexes as opposed to free Hp (Kristiansen et al., 2001; Schaer et al., 2002; Vallelian et al., 2010). Figure 1A illustrates that after a short *in vitro* culture period of 36 h only minimal fluorescent dog Hb:Hp is taken up by dog macrophages. In contrast strong complex endocytosis can be detected when the cells were pretreated with dexamethasone at a concentration of 2.5 × 10^−7^ M. We have previously shown that this treatment regimen induces Hb:Hp uptake capacity in human PBMC derived macrophages (Schaer et al., 2002). Comparable low endocytosis in untreated cells and strong glucocorticoid stimulation of endocytosis was also observed in similar experiments using human instead of dog Hp (not shown). Figure 1B shows quantitative dog Hb:Hp complex uptake data that were obtained with monocytes prepared from three different Beagles. Figure 2A illustrates the minimal uptake of free fluorescent human and dog Hp by macrophages that have been differentiated by M-CSF and dexamethasone for 4 days to fully induce the clearance system. In contrast, both dog and human Hp are strongly endocytosed in the presence of equimolar concentrations of non-fluorescent human Hb (identical results were obtained with dog Hb, data not shown). The Hb:Hp complexes accumulate in the perinuclear intracellular compartment of the dog macrophages (Figure 2B). This observation supports the specificity of the dog clearance system for Hb:Hp complexes. Figure 2C provides quantitative endocytosis data that have been obtained with macrophages prepared from five different beagle dogs. Comparison of the background corrected fluorescence signals may suggest that the dog Hb:Hp complex is more efficiently taken up than the human Hb:Hp complex. However, without more specific label free receptor-ligand interaction studies it is impossible to discriminate whether the relatively small difference reflects a true difference in Hb:Hp endocytosis or whether it might be explained by slightly different labeling or different stability of the two labeled complexes in the lysosomal compartment of the dog macrophage. Apparently, this *in vitro* observation does not reflect different *in vivo* clearance of the two complexes.

The observation that both, dog and human Hb:Hp complexes are taken up by dog macrophages via a specific clearance system suggests that the dog might be an appropriate animal model to study systemic clearance of human plasma derived Hp therapeutics.

### Pharmacokinetics of dog and human Hb in beagle dogs

**Figure 3:**
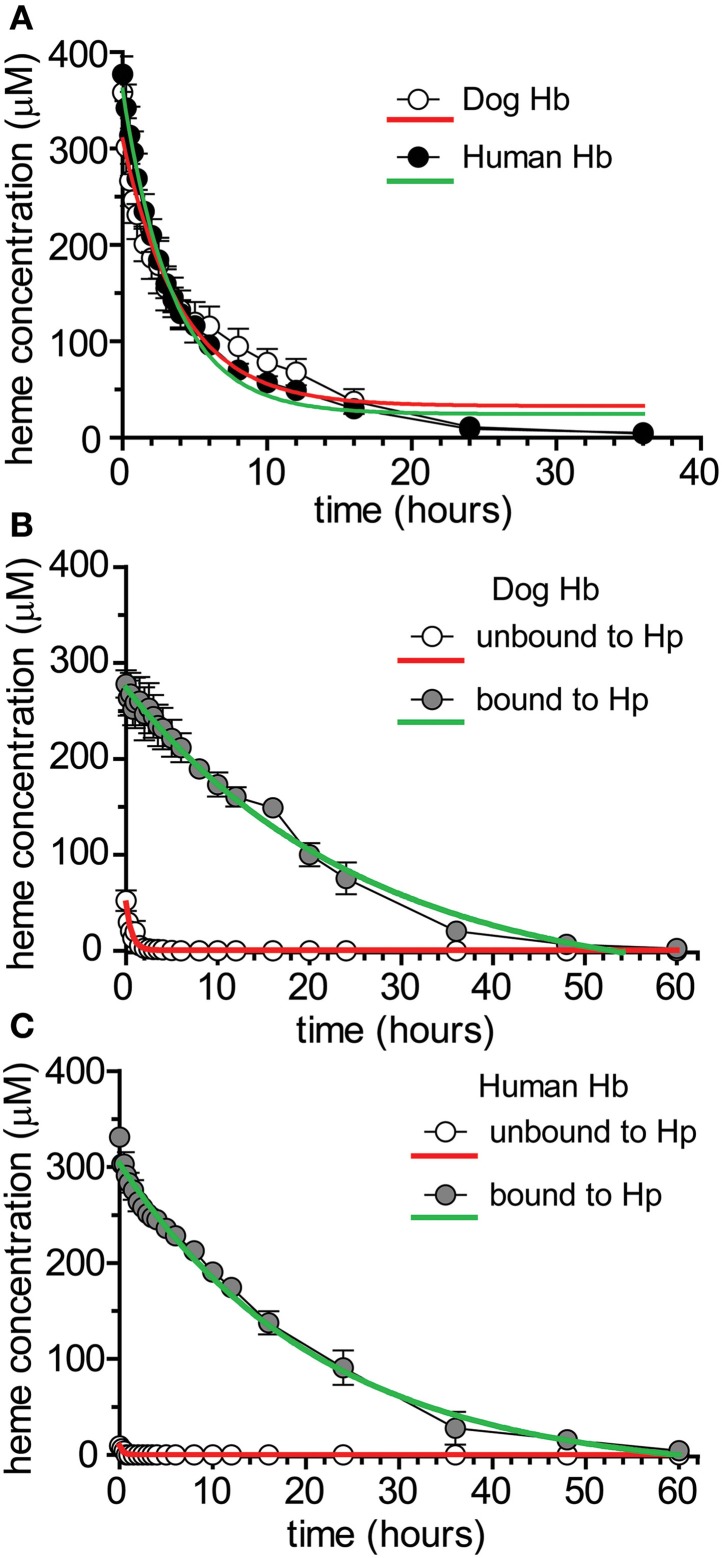
**Pharmacokinetics of dog and human Hb ± glucocorticoid up-regulation of Hb clearance systems. (A)** Mean ± SEM plasma concentration vs. time plotted for dog and human purified Hb administered to beagle dogs (*n* = 4/treatment). Data are fitted to a single exponential decay (red and green traces) and represent total heme bound to Hb + Hb:Hp. Pharmacokinetic parameter estimates are shown in Table 1. **(B)** Mean ± SEM plasma concentration vs. time plotted for prednisone pre-treated Beagle dogs administered purified dog Hb (*n* = 6). Data represent unbound and Hp bound concentrations in plasma. Data are fitted to a single exponential decay and represent total heme bound to Hb + Hb:Hp. Pharmacokinetic parameter estimates are shown in Table 2. **(C)** Mean ± SEM plasma concentration vs. time plotted for prednisone pretreated Beagle dogs administered purified human Hb (*n* = 6). Data represent unbound and Hp bound concentrations in plasma. Data are fitted to a single exponential decay (red and green traces) and represent total heme bound to Hb + Hb:Hp. Pharmacokinetic parameter estimates are shown in Table 2.

To characterize potential differences in the pharmacokinetics of dog and human Hb following acute exposures in Beagles, purified dog Hb [4 g dose (*n* = 4)] and human Hb [4 g dose (*n* = 4)] was administered by bolus intravenous administration through the jugular vein. Blood sampling was performed to cover the range of plasma concentrations from Cmax until the absence of Hb in plasma. Table 1 shows the mean ± SEM. pharmacokinetic parameter estimates for dog and human Hb. Figure 3A shows the plot of mean ± SEM plasma concentration vs. time data used to derive the pharmacokinetic parameters in Table 1. Data are fit to a single exponential decay and do not consider the differential kinetics of free Hb and Hb bound to the endogenous dog Hp. Both dog and human Hb demonstrate comparable pharmacokinetic parameters.

**Table 1:** **Pharmacokinetic parameters of dog and human hemoglobin (Hb) (mean ± SEM) in Beagles**.

**Hemoglobin [heme]**	***C*_max_(μmol/L)**	AUC_0–infinity_(h*μmol/L)	**CL (L/h)**	***Vc* (L)**	***t*_1/2_(h)**
Dog Hb (total)	358.3 ± 43.2	2075 ± 391	0.1353 ± 0.0265	0.7359 ± 0.106	3.936 ± 0.361
Human Hb (total)	377.2 ± 18.3	1929 ± 148	0.1319 ± 0.0100	0.667 ± 0.035	3.537 ± 0.148

### Pharmacokinetics of dog and human Hb following glucocorticoid up-regulation of endogenous Hb clearance systems

To characterize the differences in the pharmacokinetics of free Hb and Hb bound to Hp (Hb:Hp complex) we repeated the above described study in glucocorticoid treated Beagles, which have maximally induced endogenous Hp. Dogs were pre-treated with prednisone (50 mg, twice daily, 3 days) based on our previous studies (Boretti et al., 2009) and the studies of others (Martinez-Subiela et al., 2004). On day 4 dogs were randomized to groups receiving dog Hb [4 g dose (*n* = 6)] or human Hb [4 g dose (*n* = 6)] administered by bolus intravenous administration through the jugular vein. Blood sampling was performed to cover the range of plasma concentrations from Cmax until the absence of Hb:Hp in plasma. Table 2 shows the mean ± SEM pharmacokinetic parameter estimates for dog and human Hb unbound and bound to endogenous Hp. Figure 3B shows the plot of mean ± SEM plasma concentration vs. time data for dog Hb, while Figure 3C shows the plot of mean ± SEM plasma concentration vs. time data for human Hb used to derive the pharmacokinetic parameters shown in Table 2. Data are fit to a single exponential decay and demonstrate that both dog and human Hb are comparably bound to the glucocorticoid enhanced endogenous dog Hp *in vivo*. Pharmacokinetic parameters of dog and human Hb not bound to Hp demonstrate a low exposure (Cmax and AUC), rapid CL and large Vc that expands beyond the central compartment. This observation may be due to renal distribution of unbound Hb, as hemoglobinuria was observed following Hb exposure in the absence of Hp up-regulation or Hp administration, consistent with previous studies (Boretti et al., 2009; Lipiski et al., 2013). Both dog and human Hb bound to Hp demonstrate comparable exposures (Cmax and AUC) and significantly reduced CL compared to unbound Hb. The Vc reflects the volume of the central compartment in the Beagle and demonstrates that the Hb:Hp complex remains sequestered within the circulating blood volume. The t_1/2_ of the endogenous dog Hp complex with dog or human Hb is highly comparable. The small differences in Cmax and Vc observed at low plasma concentrations of unbound dog and human Hb may suggest minor differences in Hb binding immediately after dosing. However, the contribution to overall pharmacokinetic exposure was not changed.

**Table 2:** **Pharmacokinetic parameters of dog and human Hb (+) prednisone (mean ± SEM) in Beagles**.

**Hemoglobin [heme]**	***C*_max_ (μmol/L)**	AUC_0–infinity_ (h*μmol/L)	**CL (L/h)**	***Vc* (L)**	***t*_1/2_(h)**
Dog Hb (unbound)	52.15 ± 10.6	9.985 ± 2.59	35.73 ± 9.79	6.051 ± 1.25	0.2006 ± 0.0787
Dog Hb (bound)	277.9 ± 14.3	4798 ± 405	0.05405 ± 0.00463	0.912 ± 0.0493	12.05 ± 1.04
Human Hb (unbound)	20.1 ± 5.51	7.740 ± 2.23	48.40 ± 15.1	18.11 ± 5.46	0.2657 ± 0.0185
Human Hb (bound)	353.7 ± 18.0	5457 ± 185.2	0.03538 ± 0.00305	0.7562 ± 0.0172	11.45 ± 0.527

*(Unbound) and (bound) to Hp*.

Collectively, these data suggest differences in distribution, exposure and clearance of free Hb and the Hb:Hp complex, and demonstrate that dog and human Hb follow similar pharmacokinetics in Beagles when bound to Hp.

### Pharmacokinetics of human Hb following administration of dimeric or multimeric Hp

**Figure 4:**
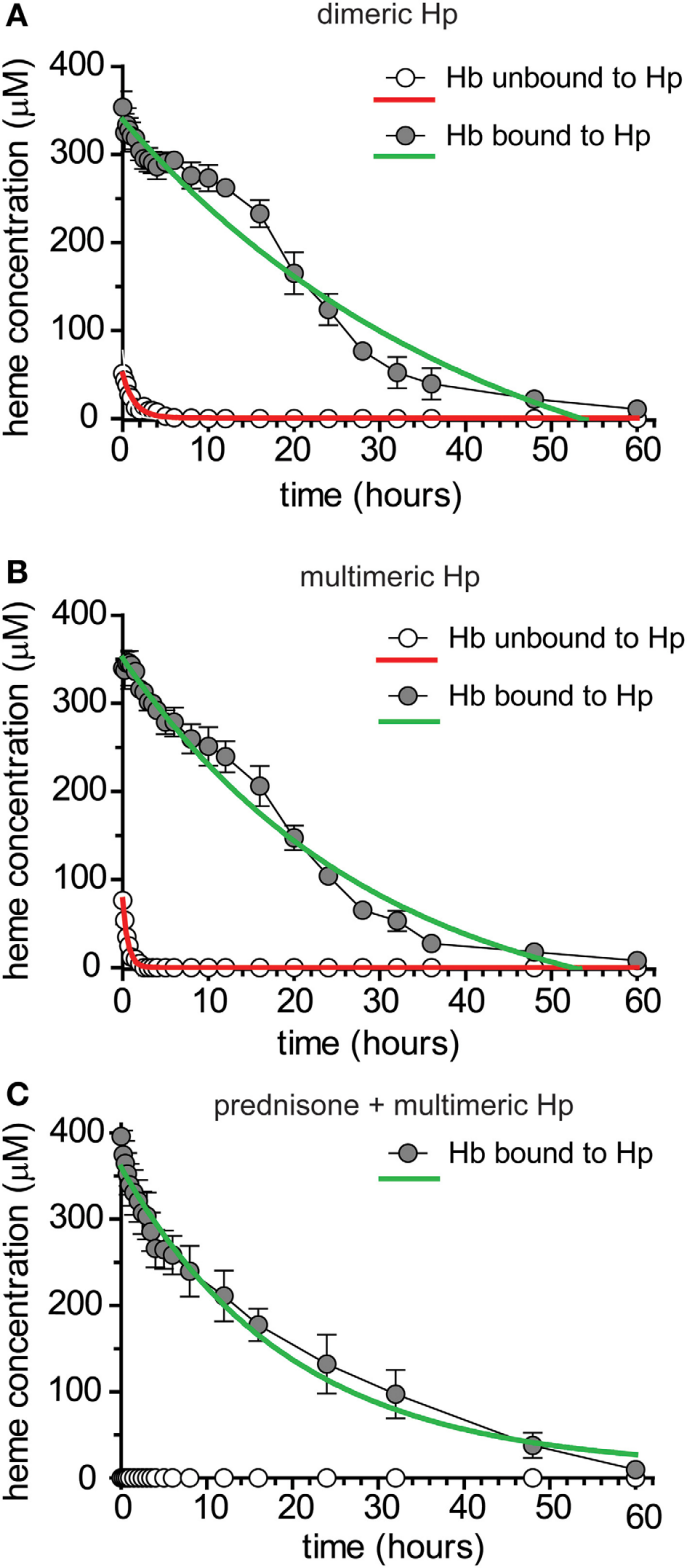
**Pharmacokinetics of human Hb following administration of dimeric or multimeric Hp ± glucocorticoid up-regulation of Hb clearance systems. (A)** Mean ± SEM plasma concentration vs. time plotted for human purified Hb + human purified dimeric Hp administered to beagle dogs (*n* = 6). Data are fitted to a single exponential decay (red and green traces) and represent total heme bound to Hb + Hb:Hp. Pharmacokinetic parameter estimates are shown in Table 3. **(B)** Mean ± SEM plasma concentration vs. time plotted for human purified Hb + human purified multimeric Hp administered to beagle dogs (*n* = 6). Data are fitted to a single exponential decay (red and green traces) and represent total heme bound to Hb + Hb:Hp. Pharmacokinetic parameter estimates are shown in Table 3. **(C)** Mean ± SEM plasma concentration vs. time plotted for prednisone pre-treated Beagle dogs administered human purified Hb + human purified multimeric Hp (*n* = 6). Gray circles represent Hb:Hp complex and open circles represent unbound Hb. No unbound Hb was detected, as result data is set to zero. Data are fitted to a single exponential decay (red and green traces) and represent total heme bound to Hb + Hb:Hp. Pharmacokinetic parameter estimates are shown in Table 4.

In a next study we characterized the pharmacokinetics of Hb following administration of dimeric (primarily 1-1) and multimeric (2-1 and 2-2) human plasma derived Hp. These data should identify potential Hp phenotypic differences in exposure, distribution and clearance after acute dosing. Furthermore, these data allowed us to test for equivalence of dog and human Hb:Hp clearance in Beagles. Dogs were randomized to groups administered 4 g human Hb followed by dimeric human Hp, 4 g dose (*n* = 6) or 4 g human Hb followed by multimeric human Hp, 4 g dose (*n* = 6). All proteins were administered by bolus intravenous administration through the jugular vein. Blood sampling was performed to cover the range of plasma concentrations from Cmax until the absence of Hb:Hp in plasma. Table 3 shows the mean ± SEM pharmacokinetic parameter estimates for free human Hb and the different Hb:Hp complexes that formed *in vivo*. Figure 4A shows the plot of mean ± SEM plasma concentration vs. time data for Hb following dimeric Hp dosing, while Figure 4B shows the plot for mean ± SEM plasma concentration vs. time data for Hb following multimeric Hp dosing. Derived pharmacokinetic parameters are shown in Table 3. Data are fit to a single exponential decay and demonstrate that Hb is comparably bound to dimeric and multimeric Hp *in vivo*. Pharmacokinetic parameters of Hb bound to dimeric and multimeric Hp demonstrate a comparable exposure (Cmax and AUC), significantly slower CL and a contracted Vc compared to unbound Hb. The t_1/2_ of the Hb:Hp dimeric and multimeric complex is highly comparable in Beagles. These data support that dimeric and multimeric Hb:Hp complexes demonstrate similar *in vivo* pharmacokinetics. Overall, the pharmacokinetic parameters of both human Hp complexes are highly comparable to the parameter estimates for dog Hb:Hp complexes (Tables 2, 3). The plasma concentration vs. time curves shown in Figures 4A,B demonstrate two distinct phases. We speculate that this observation may be the result of CD163 availability on the surface of monocytes and macrophages over the duration of the experiment.

**Table 3:** **Pharmacokinetic parameters of Hb bound to dimeric and multimeric Hp (mean ± SEM) in Beagles**.

**Hemoglobin [heme]**	***C*_max_(μmol/L)**	AUC_0–infinity_ (h*μmol/L)	**CL (L/h)**	***Vc* (L)**	***t*_1/2_(h)**
**DIMERIC Hp**					
Human Hb (unbound)	54.14 ± 4.95	78.63 ± 16.9	3.711 ± 0.862	5.022 ± 0.463	1.034 ± 0.139
Human Hb (bound)	353.7 ± 18.0	7234 ± 656	0.03538 ± 0.00306	0.7124 ± 0.0368	14.15 ± 0.956
**MULTIMERIC Hp**					
Human Hb (unbound)	78.67 ± 2.82	64.89 ± 17.8	5.156 ± 1.30	3.193 ± 0.109	0.5640 ± 0.132
Human Hb (bound)	322.1 ± 23.6	6105 ± 593	0.04297 ± 0.00527	0.7939 ± 0.0610	13.14 ± 0.983

*(Unbound) and (bound) to Hp*.

### Pharmacokinetics of Hb:Hp complexes in glucocorticoid treated dogs

*In vitro* and *in vivo* stimulation of human peripheral blood monocyte CD163 by glucocorticoids leads to increased uptake of Hb:Hp complexes and we have demonstrated a comparable response of dog monocytes. To test the contribution of the glucocorticoid inducible CD163 pool on *in vivo* pharmacokinetics we evaluated the clearance of Hb:Hp complexes in animals that have been pre-treated with 50 mg prednisone, twice daily for 3 consecutive days, which corresponds to a dose of 7–10 mg/kg/day. We have previously shown in human patients with acute inflammatory and autoimmune diseases that comparable immunosuppressive glucocorticoid treatment protocols stimulate strong CD163 expression and Hb:Hp endocytosis capacity in peripheral blood monocytes (Vallelian et al., 2010). Six dogs were dosed with human Hb (4 g) and multimeric human Hp (4 g). Blood sampling was performed to cover the range of plasma concentrations from Cmax until the absence of Hb:Hp in plasma. Table 4 shows the mean ± SEM pharmacokinetic parameter estimates for Hb unbound and bound to Hp. Figure 4C shows the plot of mean ± SEM plasma concentration vs. time data for Hb in the unbound and Hp bound forms used to derive pharmacokinetic parameters shown in Table 4. Data are fit to a single exponential decay and demonstrate that Hb is entirely bound to Hp *in vivo*. This is the result of both high endogenous Hp induced by prednisone and exogenously administered human Hp. Pharmacokinetic values for Hp bound Hb show comparable values in all studies regardless of the Hp species and demonstrate comparable exposures (Cmax and AUC) and rates of CL for untreated and glucocorticoid-treated dogs (Tables 2–4). Collectively, the data demonstrates that glucocorticoid stimulation of CD163 *in vivo* does not contribute more rapid clearance of Hb:Hp. The explanation for this observation is likely that the large pool of resident macrophages in the liver and spleen constitutively express high levels of CD163, which cannot be further stimulated by glucocorticoid administration. Compared to this large pool of CD163^+^ resident spleen and liver macrophages, the highly glucocorticoid responsive pool of peripheral blood monocytes is small and appears to provide a minor contribution to systemic Hb:Hp clearance. The predominant distribution of CD163^+^ monocytes and macrophages to liver and spleen is demonstrated in both rodent and non-rodent species (Granfeldt et al., 2013).

**Table 4:** **Pharmacokinetic parameters Hb bound to multimeric Hp (mean ± SEM) following prednisone treatment in Beagles**.

**Hemoglobin [heme]**	***C*_max_(μmol/L)**	AUC_0–infinity_(h*μmol/L)	**CL (L/h)**	***Vc* (L)**	***t*_1/2_(h)**
Human Hb (unbound)	—	—	—	—	—
Human Hb (bound)	397.0 ± 33.6	6534 ± 100	0.04185 ± 0.00587	0.6469 ± 0.0511	12.17 ± 0.157

*(Unbound) and (bound) to Hp,—no free (unbound) Hb detected*.

### Plasma profiles of bound Hb:Hp complexes

**Figure 5:**
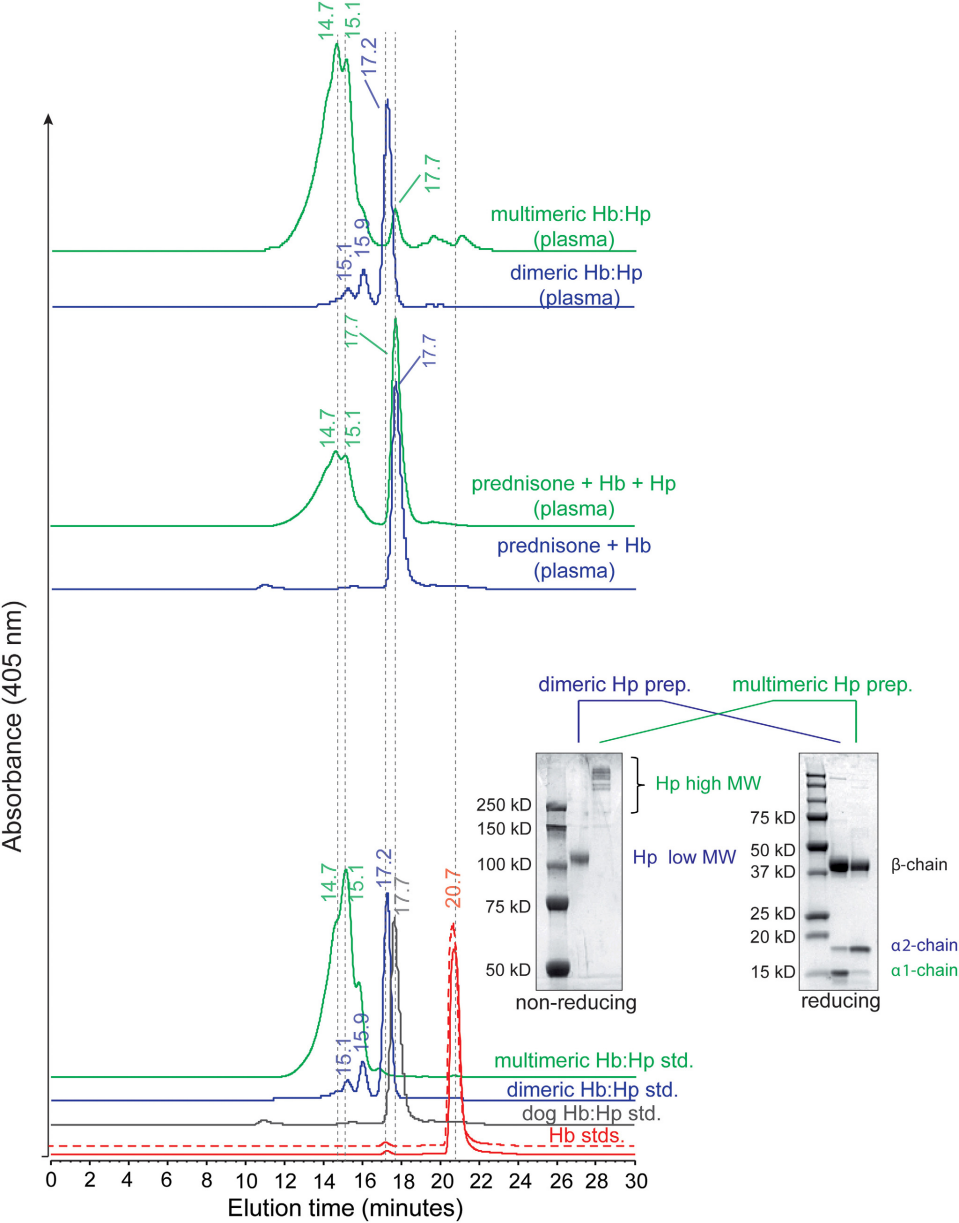
**Plasma profiles of bound Hb:Hp complexes. (Bottom panel)** SEC elution profiles of standard human Hb (solid red line), dog Hb (dashed red line) and Hb:Hp complexes. Bottom panel inset shows gel electrophoresis of dimeric and multimeric Hp preparations under non-reducing and reducing conditions. **(Middle panel)** shows representative plasma samples from prednisone pre-treated animals following Hb exposure (±) multimeric Hp. **(Top panel)** shows the distribution of Hb bound to Hp after dimeric and multimeric human Hp dosing.

Figure 5 (bottom panel) shows the SEC elution profiles of standard Hb and Hb:Hp complexes. Human and dog Hb demonstrate an elution time of 20.7 min. Dog Hb:Hp demonstrates an elution time of 17.7 min. Dog Hp is entirely in the dimeric form, but likely differs from human dimeric Hp in terms of the extent and distribution of glycosylation sites. This is observed in the chromatographic elution profile of dimeric human Hb:Hp, which elutes approximately 0.5 min faster than Hb bound dog Hp and indicates a slightly larger molecular size for the human Hb:Hp complex. Multimeric human Hp shows a broad elution profile between 12 and 16 min and no dimeric Hp phenotype. Figure 5 (bottom panel, inset) shows a gel electrophoresis of dimeric and multimeric Hp preparations under non-reducing and reducing conditions. The non-reducing gel shows the phenotype specific dimer-polymer distribution of the Hp products used in our studies. The reducing gel shows the presence of primarily α1 globin in dimeric Hp and α2 globin in multimeric Hp preparations. Figure 5 (middle panel) shows representative plasma samples from prednisone pre-treated animals following Hb exposure ± multimeric Hp. The elution time peak at 17.7 min represents prednisone induced dog Hp bound to Hb in both chromatograms (prednisone ± Hp). The elution time of multimeric Hp:Hp is observed with primary peaks at 14.7 and 15.1 min. The smaller area of the multimeric Hb:Hp peak is consistent with the large area of the dimeric dog Hp following prednisone pre-treatment. Figure 5 (top panel) shows the distribution of Hb bound to Hp after dimeric and multimeric human Hp dosing. Samples show elution times of Hp bound Hb consistent with purified standards. Samples represent the 1 h collection time point.

## Discussion

### Primary findings

Here we present *in vitro* data, which suggests that the function and fundamental regulatory pathways of the human and dog macrophage clearance system for Hb:Hp complexes are comparable. These *in vitro* observations strongly indicate that human and dog demonstrate similar pharmacologic and pharmacokinetic responses to intravascular Hb:Hp exposure and that dog may, therefore, be an appropriate model for the pre-clinical characterization of Hp therapeutics.

We provide the first pharmacokinetic parameter estimates of cell free Hb and its modulation by Hp therapeutics in a non-rodent model. The data reflect a drastically reduced Vc of the complex compared to free Hb, increased exposures (Cmax and AUC) and significantly reduced CL with a t_1/2_ of approximately 12 h. These estimates were not significantly different for the human and dog proteins, respectively. These data support the concept whereby a major protective function of Hp is provided by a limited expansion of the central compartment volume (Vc) and as a result decreased tissue compartmentalization of free Hb.

Our *in vivo* pharmacokinetic studies suggest two additional key findings.

First, the pharmacokinetics of Hp bound Hb following the administration of dimeric and multimeric plasma derived human Hp demonstrate comparable pharmacokinetic parameter estimates. These data suggest that both Hb bound to dimeric and multimeric Hp undergo similar handling by Beagle dog Hb clearance pathways. Earlier *in vitro* studies have provided conflicting data on differential uptake and post-endocytic processing of dimeric and multimeric Hb:Hp complexes by CD163 (Asleh et al., 2003, 2014; Lipiski et al., 2013). Our *in vivo* data suggest that in a setting of acute therapeutic Hp administration the phenotype composition of a product will most likely not determine clearance and cumulative exposure. The endogenous dog Hp is only expressed in a dimeric composition without other Hps similar to the 2-1 or 2-2 phenotypes found in humans (Mominoki et al., 1995). Moreover different glycosylation patterns exist between dog and human Hp (Mominoki et al., 1995) and this could potentially contribute to altered CD163 receptor interactions. Given these known differences it is notable that highly comparable exposure times and clearance rates are observed in Beagle dogs with Hp proteins novel to the species. This observation supports that the Hb:Hp clearance system is conserved among non-rodent animal species and human.

Second, data from Figures 1, 2 clearly demonstrate that the function and regulation of the dog macrophage Hb:Hp clearance system is comparable to the human system. Both systems are specific to the Hb:Hp complex as opposed to free Hp (Kristiansen et al., 2001). This specificity of the clearance system for the complex helps to preserve high circulating Hp concentrations. Additionally, the expression of the clearance system as well as its down-stream effector pathways is strongly stimulated by glucocorticoid treatment of peripheral blood monocytes (Schaer et al., 2002, 2008). In human, the glucocorticoid stimulation of Hb:Hp uptake triggers a complex differentiation pathway that promotes monocyte differentiation into an anti-oxidative and iron handling macrophage phenotype during increased extracellular Hb exposure (Vallelian et al., 2010). Glucocorticoid stimulation of Hb:Hp uptake by peripheral blood monocytes could also enhance total body Hb:Hp clearance capacity, however this has not been investigated so far. Our pharmacokinetic data suggest that glucocorticoid stimulation of the peripheral blood monocyte Hb:Hp clearance system does not significantly enhance systemic Hb clearance, which appears to be predominantly mediated by resident tissue macrophages in the liver and spleen. These cells already express maximum levels of CD163 and high HO-1 enzymatic capacity under physiologic conditions (Zwadlo et al., 1987).

### Study limitations

The shortcomings of this study are clearly that no data exists to compare dog to human in terms of pharmacokinetics of Hb:Hp complexes following exogenous administration during hemolysis. In the future human studies evaluating the pharmacokinetics of Hb:Hp will provide important information that will contribute to the appropriate dosing and potential safety concerns associated with long circulating Hb:Hp complexes if saturable pharmacokinetics are found to occur. Based on our data in dogs, Hb:Hp complexes are cleared more rapidly than in rodent species.

### Novelty

The novelty of the data presented identify the Beagle dog as relevant species that may provide a better understanding of Hb:Hp clearance in humans. These findings are applicable to recent interests in developing plasma derived Hp as a biologic therapeutic for uses in both acute and chronic hemolytic disease states and provide data to support pharmacologic and pharmacokinetic similarity for predicting human safety. The data from dogs follow single compartment pharmacokinetics with a Vc limited to the plasma compartment. The clearance from the plasma compartment is slower than for Hb however much more rapid than in rodent species (Lipiski et al., 2013). We speculate that more rapid pharmacokinetic clearance *in vivo* and the evidence of cellular clearance *in vitro* suggest that the dog may be translatable to human. The identification of a non-rodent animal model may also prevent the need for genetic modifications to humanize the clearance pathway of animal species to enhance human predictability.

### Conflict of interest statement

The authors declare that the research was conducted in the absence of any commercial or financial relationships that could be construed as a potential conflict of interest.
